# CYB5R3 overexpression preserves skeletal muscle mitochondria and autophagic signaling in aged transgenic mice

**DOI:** 10.1007/s11357-022-00574-8

**Published:** 2022-05-09

**Authors:** Sara López-Bellón, Sandra Rodríguez-López, José A. González-Reyes, M. Isabel Burón, Rafael de Cabo, José M. Villalba

**Affiliations:** 1 Departamento de Biología Celular, Fisiología E Inmunología, Universidad de Córdoba, Campus de Rabanales, Edificio Severo Ochoa, 3ª planta, Campus de Excelencia Internacional Agroalimentario, ceiA3, 14014 Cordoba, Spain; 2 Translational Gerontology Branch, National Institute On Aging, National Institutes of Health, Baltimore, MD USA

**Keywords:** Aging, Autophagy, Cytochrome *b*_5_ reductase, Mitochondria, Skeletal muscle

## Abstract

Cytochrome *b*_5_ reductase 3 (CYB5R3) overexpression activates respiratory metabolism and exerts prolongevity effects in transgenic mice, mimicking some of the salutary effects of calorie restriction. The aim of our study was to understand how CYB5R3 overexpression targets key pathways that modulate the rate of aging in skeletal muscle, a postmitotic tissue with a greater contribution to resting energy expenditure. Mitochondrial function, autophagy and mitophagy markers were evaluated in mouse hind limb skeletal muscles from young-adult (7 months old) and old (24 months old) males of wild-type and CYB5R3-overexpressing genotypes. Ultrastructure of subsarcolemmal and intermyofibrillar mitochondria was studied by electron microscopy in red gastrocnemius. CYB5R3, which was efficiently overexpressed and targeted to skeletal muscle mitochondria regardless of age, increased the abundance of complexes I, II, and IV in old mice and prevented the age-related decrease of complexes I, III, IV, and V and the mitofusin MFN-2. ATP was significantly decreased by aging, which was prevented by CYB5R3 overexpression. Coenzyme Q and the mitochondrial biogenesis markers TFAM and NRF-1 were also significantly diminished by aging, but CYB5R3 overexpression did not protect against these declines. Both aging and CYB5R3 overexpression upregulated SIRT3 and the mitochondrial fission markers FIS1 and DRP-1, although with different outcomes on mitochondrial ultrastructure: old wild-type mice exhibited mitochondrial fragmentation whereas CYB5R3 overexpression increased mitochondrial size in old transgenic mice concomitant with an improvement of autophagic recycling. Interventions aimed at stimulating CYB5R3 could represent a valuable strategy to counteract the deleterious effects of aging in skeletal muscle.

**Graphical abstract:**

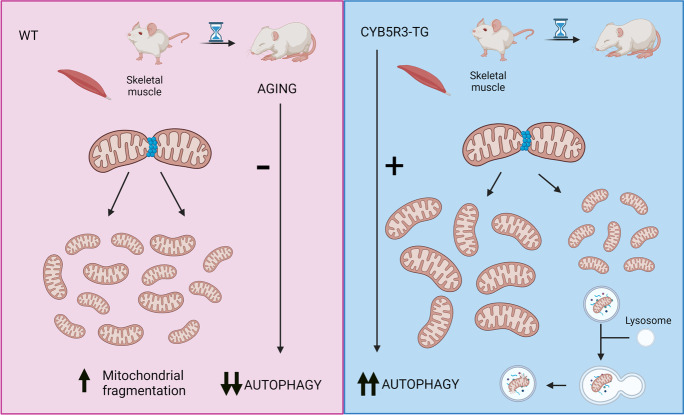

**Supplementary information:**

The online version contains supplementary material available at 10.1007/s11357-022-00574-8.

## Introduction

As a gradual time-dependent decline of the normal physiological functions, aging is considered the most important risk factor for chronic diseases [1]. The free radical theory of aging postulates that aging is caused by an increase in oxidant species production, mainly of mitochondrial origin, and a decrease of antioxidant defenses [2]. Accordingly, mitochondrial dysfunction appears as one of the best characterized hallmarks of aging [3], which points towards a key role of this organelle in understanding those processes associated with the development of senescence.

Post-mitotic tissues, as skeletal muscle, are specially affected by aging-associated mitochondrial dysfunction [4]. Skeletal muscles of most mammals contain two basic types of fibers: red fibers (RF, also known as type I or slow-twitch), which express myosin heavy chain 1 (MHC I) and have an oxidative metabolism, and white fibers (WF, also regarded as type II or fast-twitch), which express MHC II isoforms and can be further subdivided in type IIA fibers (MHC IIA, which are oxidative and are also regarded as intermediate fibers), and type IIX and IIB fibers (MHC IIX and IIB) which are glycolytic [5]. Aging produces alterations in both type I and II fibers, although they are affected differently [6]. One of the principal effects of aging on skeletal muscle is sarcopenia, whose onset is related with a decrease in mitochondrial content and functionality, highlighting the importance of the studies focused on this organelle [7].

As highly dynamic organelles, mitochondria undergo continuous cycles of fusion and fission to maintain a healthy state [8]. The mitofusins 1 and 2 (MFN-1 and MFN-2) are key proteins mediating fusion, whereas DRP-1, FIS1, and mitochondrial fission factor (MFF) are involved in fission. Damaged mitochondria undergo fission to facilitate their degradation and clearance by mitophagy, a selective form of autophagy whose principal mechanism proceeds through the PINK1/PARKIN pathway [9]. Maintenance of a healthy mitochondrial population also requires mitochondrial biogenesis, where PGC-1α interacts with NRF-1 to activate the expression of TFAM, a transcription factor involved in mitochondrial genome replication [10]. The sirtuins SIRT1 and SIRT3 are key components of the machinery that regulates mitochondrial biogenesis, antioxidant protection, and oxidative metabolism in several tissues, including skeletal muscle [11].

Nowadays, a great interest is being paid to those interventions with a potential to extend maximal longevity (lifespan) and/or to promote healthy aging (healthspan). Calorie restriction (CR) without malnutrition is the most effective intervention that extends both lifespan and healthspan in many model organisms [12]. Identification of enzymes mimicking the effects of CR is another area of great interest because they constitute potential targets in antiaging pharmacological interventions. NADH-cytochrome *b*_5_ reductase 3 (CYB5R3, EC 1.6.2.2) is a flavoprotein that catalyzes electron transfer from NADH to cytochrome *b*_5_ or to alternative electron acceptors including plasma membrane coenzyme Q and several exogenous compounds [13]. The membrane-bound isoform of CYB5R3 is attached on the cytosolic side of the mitochondrial outer membrane, endoplasmic reticulum, and plasma membrane; is ubiquitously expressed in many tissues; and participates in drug metabolism, elongation, and desaturation of fatty acids and cholesterol biosynthesis [14]. Acting in tandem with the lipophilic electron carrier coenzyme Q, CYB5R3 participates in a trans-plasma membrane redox system which protects cells against oxidants [15].

To gain further insights into the participation of CYB5R3 in the regulation of those pathways that influence the rate of aging, we generated mice overexpressing CYB5R3 (TG mice), which displayed enhanced protection against induced cancer, increased insulin sensitivity, less oxidative damage, and extended longevity [16]. Despite these positive effects, to date, no study has been set up to address how CYB5R3 overexpression modulates key pathways related with longevity in aged mice.

The aim of this work was to study how CYB5R3 overexpression affects mitochondrial morphology and function as well as autophagy in skeletal muscle from young-adult and aged TG mice. Our results demonstrate that, consistent with its prolongevity effects in mice, CYB5R3 overexpression protects mitochondria from aging-associated biochemical and structural alterations and modulates autophagic signaling in skeletal muscle.

## Methods

### Animals and diets

This study was carried out with a cohort of wild-type (WT) and CYB5R3-transgenic (TG) male mice in a C57BL/6 background that was generated as previously reported [16] (see Supplemental Methods). WT and TG mice were further separated in two age groups which were designated as Y (stating for young-adult) and O (stating for old). All animals had ad libitum access to a standard chow from weaning until they reached 3 months of age. Then, they were transferred to a purified AIN93M diet and fed for 4 (Y groups) or 21 (O groups) additional months. Once the animals reached the required age (7 or 24 months), they were sacrificed by cervical dislocation. Hind limb muscles were rapidly dissected, trimmed from fat and connective tissue, and frozen by immersion in liquid nitrogen in a buffered medium containing 10% DMSO as cryoprotectant. Tissue samples were then stored at − 80 ºC for further biochemical analysis. Gastrocnemius samples were also removed and immediately processed for electron microscopy analysis as described below. Procedures with experimentation animals were authorized by the Consejería de Agricultura, Pesca y Desarrollo Rural, Junta de Andalucía (authorization code: 20/04/2016/053).

### Preparation of tissue extracts

Hind limb skeletal muscle samples from six animals per group were homogenized in radioimmunoprecipitation assay (RIPA) buffer (50 mM Tris–HCl pH 8; 150 mM NaCl; 0.5% deoxycholate; 0.1% SDS; 1% Triton X-100; 1 mM DTT; 1 mM phenylmethylsulfonyl fluoride (PMSF); 10 μg/mL each of chymostatin, leupeptin, antipain, and pepstatin A (CLAP); and phosphatase inhibitor cocktails 2 and 3 (Sigma-Aldrich) diluted at 1/100) for 30 s using a high-performance dispersing instrument (Ultra-Turrax T25, IKA, Staufen, Germany). Homogenates were then centrifuged at 10,000 × g for 15 min at 4 ºC and the supernatants stored frozen at – 80 ºC until further analysis. Total protein was determined by the Stoscheck modification [17] of the Bradford dye-binding method [18].

### Isolation of mitochondria-enriched fractions

Hind limb skeletal muscles clean of fat and connective tissue were homogenized during 30 s at 4 ºC in ice-cold buffer containing 20 mM Tris–HCl pH 7.6, 40 mM KCl, 0.2 M sucrose, 1 mM PMSF, 10 mM EDTA, 1 mM DTT, 20 μg/μL CLAP, and phosphatase inhibitor cocktails 2 and 3 (Sigma-Aldrich) diluted at 1/100, using an electric tissue disrupter (Ultra-Turrax T25, IKA, Staufen, Germany). The homogenates were centrifuged at 420 × g for 10 min to eliminate cell nuclei and unbroken cells. Supernatants were collected and centrifuged at 6,700 × g for 10 min to obtain a mitochondria-enriched fraction in the pellet. This pellet was resuspended in 100 μl of isolation buffer and stored at − 80 ºC.

### Electrophoresis and Western blot immunodetection

Electrophoresis and electro-transference to nitrocellulose membranes were performed as described in our previous report [19] using the primary antibodies listed in Table 1 and secondary antibodies coupled to horseradish peroxidase to reveal binding sites by enhanced chemiluminescence (ClarityTM Western ECL Blotting Substrates Kit, Bio-Rad). The signal was recorded using a ChemiDoc Imaging System (Bio-Rad), and the digital images obtained were analyzed using the Image LabTM Software (Bio-Rad). Quantification data of immunostained bands were normalized to the overall image density of the corresponding lane stained with Ponceau S.

**Table 1:** Primary antibodies used in this study. The table shows the concentrations and the commercial references of each antibody. (*SC* Santa Cruz antibodies)

Primary antibodies	Dilution	Reference	Primary antibodies	Dilution	Reference
CYB5R3	1:50,000	Proteintech 10,894–1-AP	DRP-1	1:500	SC-32898
PINK1	1:1000	SC-33796	FIS1	1:500	SC-98900
PARKIN	1:100	Cell Signaling 2132	MFF	1:1000	Cell Signaling 84,580
P62	1:3000	Sigma AldrichP0067	VDAC	1:1000	SC-98708
LC3 A/B	1:1000	Cell Signaling 4108	NRF-1	1:2000	SC-33771
OxPhos rodent WB antibody cocktail*	1:4000	Life technologies 458,099	TFAM	1:1000	SC-2358
MFN-1	1:1000	SC-50330	SIRT-1	1:1000	SC-15404
MFN-2	1:500	SC-50331	SIRT-3	1:1000	SC-99143

^*^OxPhos rodent WB antibody cocktail: Complex I subunit NDUFB8 (NADH dehydrogenase (ubiquinone) 1 beta subcomplex subunit 8), Complex II subunit SDHB (Succinate dehydrogenase (ubiquinone) iron-sulfur subunit), Complex III subunit UQCRC2 (Cytochrome b-c1 complex subunit), Complex IV subunit MTCO1 (mitochondrially encoded cytochrome c oxidase I), Complex V subunit ATP5A (Complex V alpha subunit)

### Determinations of coenzyme Q levels

Lipid extraction from muscle samples and CoQ quantification by HPLC were performed as described by Fernández-del-Río et al. [20] (see Supplemental Methods).

### ATP determinations

ATP was quantified by using a luciferine/luciferase-based measurement kit (Molecular Probes). In brief, a standard reaction solution was prepared with 400 µl 20 × reaction buffer, 80 µl 0.1 M DTT, 400 µl of 10 mM d-luciferine, 2 µl of 5 mg/ml firefly luciferase, and water to a final volume of 8 ml. Samples of RIPA homogenates (3.7 µl) were mixed with 71.25 µl of standard reaction solution, and the mixtures were then transferred to a 96-well microplate for measurement of ATP bioluminescence using a Varioskan LUX microplate luminometer (Thermo Scientific). ATP concentrations were calculated from a standard curve obtained with known amounts of ATP in standard reaction solution.

### Structural and ultrastructural analysis of mitochondria

Samples of red gastrocnemius were fixed in aldehydes and embedded in epoxy resin following a standard protocol for transmission electron microscopy. A detailed procedure is included in supplementary Material and Methods. Micrographs from longitudinal and transversal sections of skeletal muscle were taken randomly at 20,000 × and used for planimetric (sectional area) and stereological analyses of mitochondria. Stereological analyses were performed to calculate numerical profile density (Na), which measures the number of mitochondria or autophagic figures per µm^2^ of cell surface, and volume density (Vv), defined as the volume of a given subcellular structure per volume unit of the cell (expressed in µm^3^/µm^3^). To this purpose, we followed the point counting method of Weibel [21] by superposing the pictures with a simple square lattice with 0.4-µm separation between points. All measurements were performed using ImageJ software (NIH).

### Statistical analysis

Data were expressed as mean ± SEM. Normality of the values was verified using the Kolmogorov–Smirnov normality test. The means were compared by two-tailed Student’s *t* test, whereas global effects of age or genotype were assessed by two-way ANOVA. In case data did not pass the normality test, the nonparametric two-tailed Mann–Whitney test was followed. Statistically significant differences that were only evidenced by one-tailed tests were annotated as trends (*t*) in the corresponding plot. Differences in mitochondrial size among experimental groups were evaluated by frequency distribution analysis of at least 1000 mitochondria profiles from each group. Size distributions were compared by means of the nonparametric Kolmogorov–Smirnov test. Significant differences were expressed as follows: *(*p* < 0.05), **(*p* < 0.01), ***(*p* < 0.001), and ****(*p* < 0.0001). All statistical analyses and graphics were performed using GraphPad Prism 8 (GraphPad Software Inc., San Diego, CA).

## Results

### CYB5R3 overexpression is sustained in old TG mice.

**Fig. 1:**
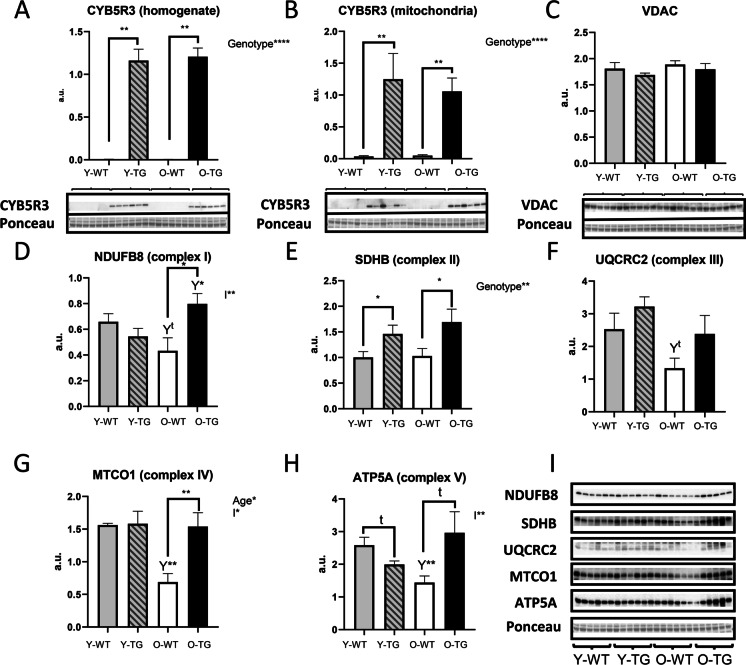
Expression levels of CYB5R3 polypeptide, VDAC, and electron transport chain complexes quantified in skeletal muscle from young/adult and old mice of WT and TG genotypes. **A**, **B** depict CYB5R3 levels in homogenates and enriched mitochondrial fractions respectively. **C** shows the levels of VDAC in homogenates. In **A**, **B**, and **C**, antibody- and Ponceau S-stained western blots are included below the graph (a.u. arbitrary units). **D** to **H** depict the quantification of representative subunits of the five ETC complexes, and the corresponding antibody- and Ponceau S-stained western blots are shown in **I**. Asterisks or t symbol connecting two bars refer to the significance of differences due to CYB5R3 overexpression (WT *vs*. TG) within a given age group. *Y* symbol (accompanied by t or asterisks) above a bar denotes the statistical significance of differences between age groups (Y *vs*. O) within a given genotype. “Genotype” denotes a global effect of CYB5R3 overexpression independently of age, “Age” indicates a global effect of aging regardless genotype, and “I” represents the existence of genotype × age interaction. Depicted data are mean ± SEM of 6 replicates

We recently demonstrated that CYB5R3 polypeptide levels increase dramatically in skeletal muscle homogenates from young-adult TG mice in comparison with their WT counterparts [14]. It is however unknown if this pattern is altered by aging, and if overexpressed CYB5R3 is efficiently targeted to skeletal muscle mitochondria. Thus, we measured the amounts of CYB5R3 polypeptide not only in whole extracts, but also in enriched mitochondrial fractions that had been isolated from hind limb skeletal muscle of both WT and TG mice at two age points: 7 months (young-adult; Y-WT and Y-TG groups, respectively) and 24 months (old; O-WT and O-TG groups, respectively). As depicted in Fig. 1A, an identical pattern of CYB5R3 overexpression was observed in TG mice regardless of age. Moreover, CYB5R3 was similarly enriched in mitochondria isolated from TG mice at the two age points (Fig. 1B). Since conditions to reveal and quantify the levels of CYB5R3 polypeptide in samples obtained from TG mice made the protein almost undetectable in samples derived from WT mice, particularly in the case of total homogenates, we also overexposed these western blots to be able to quantify the signals from WT while saturating those from TG mice and found that aging led to an increase of CYB5R3 polypeptide, both in total homogenates and in enriched mitochondrial fractions (Supplemental Figs. 1A and B).

### CYB5R3 overexpression increases the levels of mitochondrial complexes in old mice and counteracts their aging-associated decrease

We next studied the impact that age and/or CYB5R3 overexpression imposed on the levels of electron transport chain (ETC) complexes of the inner mitochondrial membrane (Fig. 1D–I), and on the outer mitochondrial membrane protein VDAC (porin; Fig. 1C). By using an antibody cocktail against representative subunits of the five ETC complexes, we evidenced that aging produced significant decreases in complexes IV (Fig. 1G) and V (Fig. 1H) in WT mice, and a trend towards a decrease of complexes I and III was also observed (Fig. 1D and F). As reported in our previous publication [14], CYB5R3 overexpression led to an increase of complex II in young-adult mice (Fig. 1E). Of note, the impact of CYB5R3 overexpression was much more pronounced at old age, since generalized increases of ETC complexes were observed in O-TG in comparison with O-WT group, which were statistically significant for complexes I (Fig. 1D), II (Fig. 1E), IV (Fig. 1G), and V (Fig. 1H). Moreover, the aging-related decrease of complexes I, IV, and V was completely abated by CYB5R3 overexpression (Fig. 1D, G, and H). Unlike the observed alterations in the abundance of ETC complexes, no changes in VDAC attributable to either age or to genotype were found in skeletal muscle homogenates (Fig. 1C).

### CYB5R3 overexpression prevents the age-related decline in ATP, but not in coenzyme Q and mitochondrial biogenesis markers

**Fig. 2:**
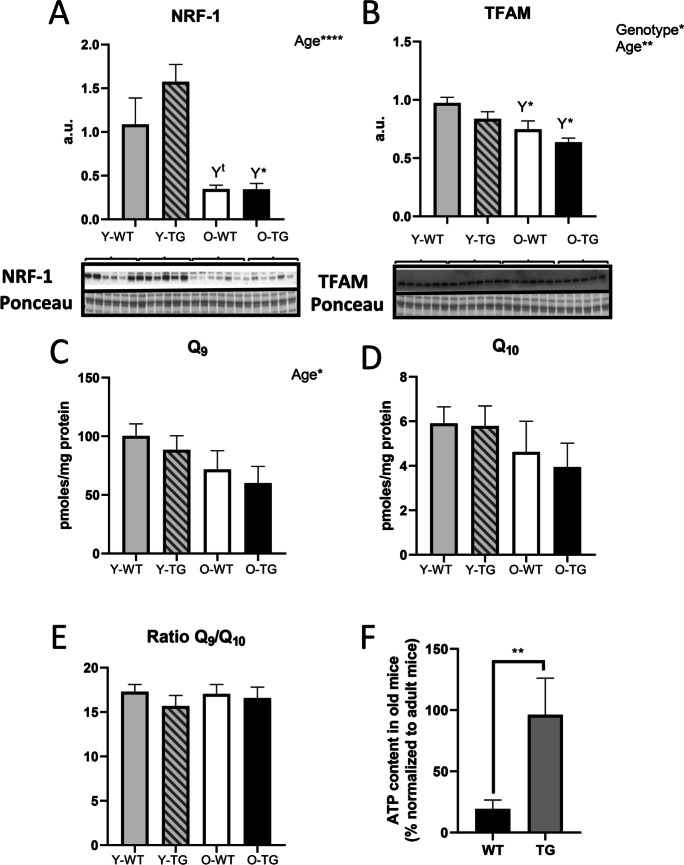
Mitochondrial biogenesis markers: NRF-1 (**A**) and TFAM (**B**); levels of coenzyme Q_9_ (**C**), coenzyme Q_10_ (**D**), and coenzyme Q_9_/Q_10_ ratio (**E**); and ATP content (**F**). *Y* symbol (accompanied by t or asterisks) above a bar denotes the statistical significance of differences between age groups (Y *vs*. O) within a given genotype. Asterisks without a letter connecting two bars refer to statistically significant differences between these groups. “Genotype” indicates a global effect of CYB5R3 overexpression independently of age, and “Age” indicates a global effect of aging regardless genotype. Antibody- and Ponceau S-stained western blots are included below the corresponding graph in **A** and **B**. Data are shown as mean ± SEM of 6 replicates (a.u. arbitrary units)

We evidenced a consistent effect of aging decreasing both TFAM and NRF-1 irrespective of genotype, the decline being particularly striking for NRF-1 (Fig. 2A and B). We also quantified coenzyme Q levels since this electron carrier and antioxidant has been shown to decrease with aging specifically in skeletal muscle although not in other tissues [22]. In accordance with this previous research, we evidenced in our model that aging produced a decrease of coenzyme Q_9_ independently of genotype (Fig. 2C), although changes in coenzyme Q_10_ did not reach statistical significance (Fig. 2D), without any modification in the ratio between coenzyme Q isoforms (Fig. 2E). Changes in total coenzyme Q paralleled those of coenzyme Q_9_ (Supplemental Fig. 2A), which agrees with this one being the predominant coenzyme Q isoform in rodent tissues. However, as also found for TFAM and NRF-1, CYB5R3 overexpression did not affect these age-related declines (see Fig. 2C–E, Supplemental Fig. 2A). Interestingly, the content of skeletal muscle ATP was significantly decreased in aged WT mice in comparison with young-adult mice of the same genotype, and this decrease was prevented by CYB5R3 overexpression (Fig. 2F).

### SIRT1 and SIRT3 levels are modulated by aging and CYB5R3 overexpression

**Fig. 3:**
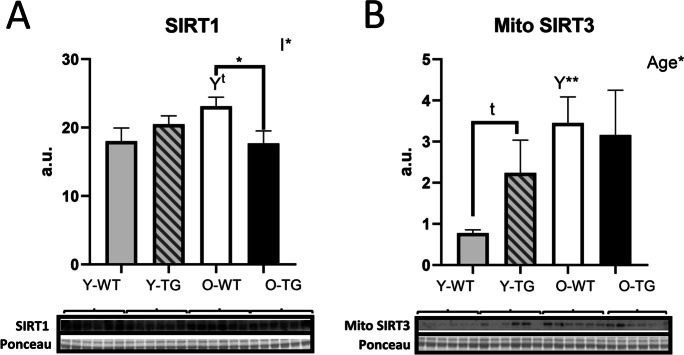
Abundance of SIRT1 (**A**) and cleaved (mitochondrial) SIRT3 (**B**). “*Age*” indicates a global effect of aging regardless genotype. Asterisks without a letter connecting two bars refer to statistically significant differences due to CYB5R3 overexpression (WT *vs*. TG) within a given age group. *Y* symbol (accompanied by t) above a bar denotes the statistical significance of differences between age groups (Y *vs*. O) within a given genotype. “Age” indicates a global effect of aging regardless genotype. “I” represents the existence of genotype × age interaction. Antibody- and Ponceau S-stained western blots are included below the corresponding graph in **A** and **B**. Data are shown as mean ± SEM of 6 replicates (a.u. arbitrary units)

We wanted to study how aging and/or CYB5R3 overexpression affected expression levels of SIRT1 and SIRT3 because these sirtuins play prominent roles in the regulation of mitochondrial biogenesis and function in skeletal muscle [11]. For SIRT3, we measured both the cleaved (mitochondrial) and full-length (nuclear) species. Changes of SIRT1 with aging and/or CYB5R3 overexpression were not striking, but those of mitochondrial SIRT3 were more noteworthy. For SIRT1, we evidenced a trend towards an increase in O-WT compared with Y-WT, and a modest decrease in O-TG in comparison with O-WT (Fig. 3A). In the case of mitochondrial SIRT3, the most notorious effect was its increase with aging in mice of WT genotype (O-WT *vs*. Y-WT). A trend towards an increase due to CYB5R3 overexpression was also observed for mitochondrial SIRT3 in young-adult mice (Y-TG *vs*. Y-WT), and its levels remained high in O-TG mice, without any difference when comparing this latter group with O-WT (Fig. 3B). Alterations of the full-length SIRT3 were similar to those observed for the cleaved isoform, with an increase by aging in WT mice (O-WT *vs*. Y-WT), and a trend towards an increase by CYB5R3 overexpression in young-adult mice (Y-TG *vs*. Y-WT) (Supplemental Fig. 2B).

### CYB5R3 overexpression upregulates markers of mitochondrial fission

**Fig. 4:**
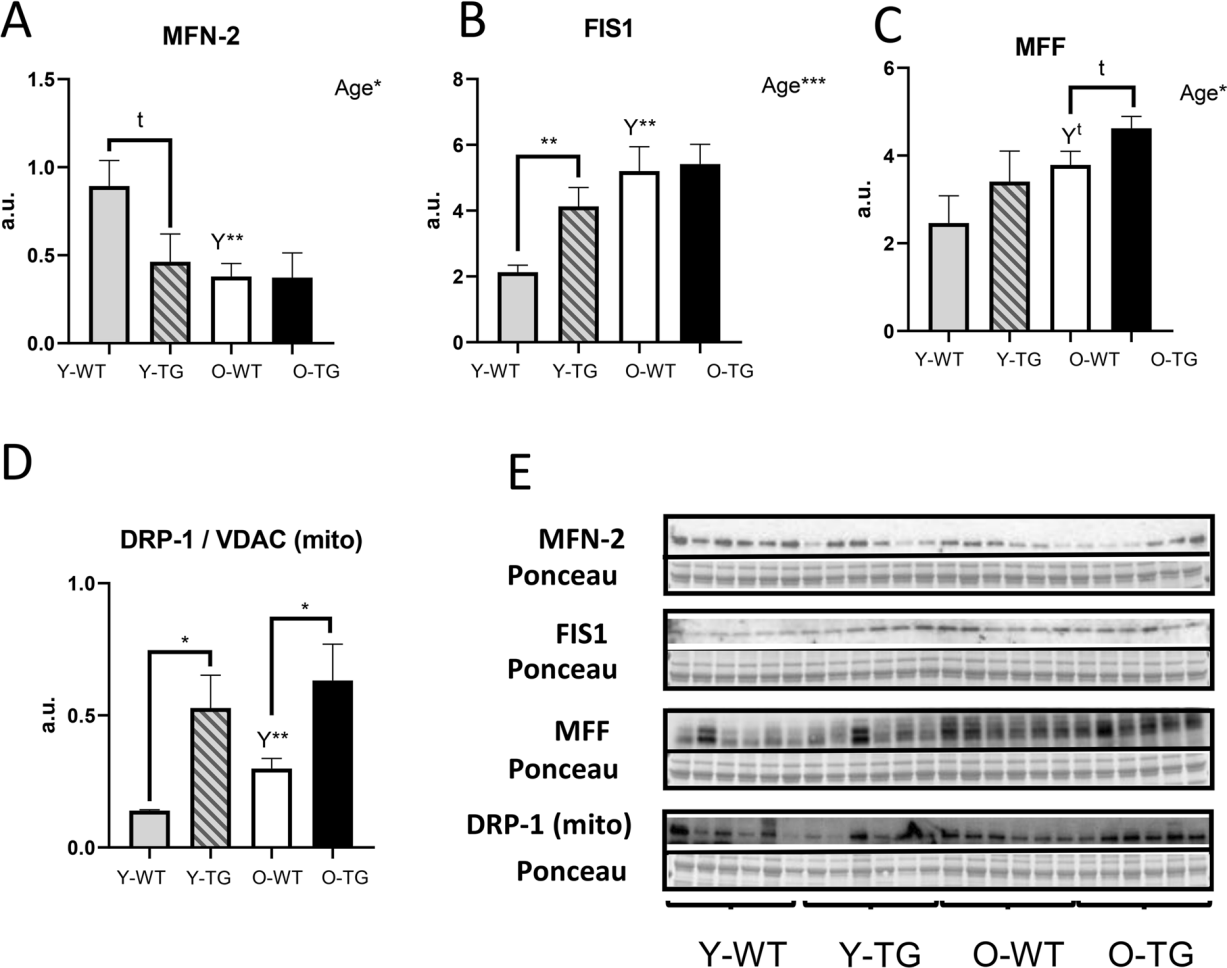
Expression levels of protein related to mitochondrial dynamics. MFN-2 (**A**), FIS1 (**B**), MFF (**C**) and the DRP-1/VDAC ratio measured in enriched mitochondrial fractions (**D**). Asterisks or t symbol connecting two bars refer to the significance of differences due to CYB5R3 overexpression (WT *vs*. TG) within a given age group. *Y* symbol (accompanied by t or asterisks) above a bar denotes the statistical significance of differences between age groups (Y *vs*. O) within a given genotype. “Age” indicates a global effect of aging regardless genotype. Antibody- and Ponceau S-stained western blots are shown in **E**. Data are shown as mean ± SEM of 6 replicates (a.u. = arbitrary units)

We next studied the effect of aging and/or CYB5R3 overexpression on protein markers of mitochondrial dynamics. MFN-1 and MFN-2 (fusion) as well as FIS1 and MFF (fission) were measured in whole extracts, while the fission-related GTPase DRP-1 was determined in isolated mitochondria fractions because DRP-1 is translocated from the cytosol to the mitochondrial outer membrane to produce fission [23]. In this latter case, data were normalized to those of VDAC to correct for variations between different mitochondrial preparations. No change due to age or genotype was observed for MFN-1 (Supplemental Fig. 3A), but MFN-2 levels showed a substantial decrease with aging in WT mice (Fig. 4A). CYB5R3 overexpression caused a downward trend in Y-TG compared with Y-WT, but no further decrease was detected in O-TG mice (Fig. 4A). Levels of FIS1 were significantly increased by aging in WT mice (Fig. 4B). Of note, CYB5R3 overexpression also produced an increase of FIS1 in Y-TG compared with Y-WT, but no further increase was observed in O-TG mice (Fig. 4B). Interestingly, changes of MFF levels with age and/or genotype resembled those of FIS1 (Fig. 4C). Mitochondria-associated DRP-1 also increased by aging in WT mice (O-WT *vs*. Y-WT) and by CYB5R3 overexpression in young-adult mice (Y-TG *vs*. Y-WT), and again, no further changes due to aging were observed in TG mice (O-TG *vs*. Y-TG) (Fig. 4D). Direct measurements of DRP-1 and VDAC in our enriched mitochondrial fractions are depicted in Supplemental Figs. 3B and C. Taken together, the changes in protein markers of mitochondrial dynamics are consistent with a prevalence of fission in O-WT and in Y-TG, without a further increase of this process in O-TG mice.

### CYB5R3 overexpression and aging alters mitochondrial morphology and abundance in WFs

The analysis of mitochondrial protein markers reported above seems to indicate the existence of adaptations that could modulate mitochondrial respiration and fusion/fission dynamics with aging and/or CYB5R3 overexpression. Thus, we carried out a study by transmission electron microscopy to confirm whether these alterations were also translated to mitochondrial ultrastructure.

**Fig. 5:**
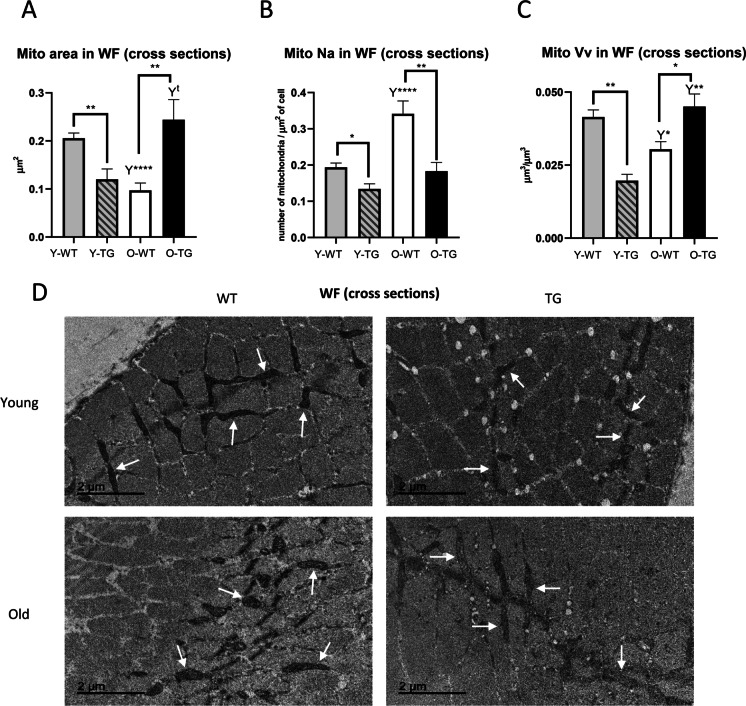
Morphometric characteristics of white fibers (WFs) mitochondria in cross-sections of gastrocnemius muscle from young-adult and old mice: mitochondrial area (**A**), numerical profile density (Na) (**B**), and volume density (Vv) (**C**). **D** shows representative electron microscopy micrographs of each group. Arrows show some examples of intermyofibrillar mitochondria. Asterisks connecting two bars refer to the significance of differences due to CYB5R3 overexpression (WT *vs*. TG) within a given age group. *Y* symbol (accompanied by t or asterisks) above a bar denotes the statistical significance of differences between age groups (Y *vs*. O) within a given genotype. Data are shown as mean ± SEM of 4 animals

In WF cross-sections, we found that CYB5R3 overexpression led to a remarkable decrease of mean mitochondrial size in Y-TG compared with Y-WT mice. The size of WF mitochondria also decreased in aged WT mice but, interestingly, an increase was observed in O-TG in comparison with O-WT mice, and a trend towards an increase was also detected when comparing O-TG with Y-TG animals (Fig. 5A). The effects of aging and/or CYB5R3 overexpression on mitochondrial size were confirmed by the statistical comparison of size distributions of all measured mitochondrial profiles, which showed that aging augmented the abundance of smaller mitochondrial profiles at the expenses of the larger ones (Supplemental Fig. 4A). Distribution analysis of mitochondrial size in WF also confirmed that in Y-TG, the number of smaller mitochondrial profiles increased at the expense of the larger ones in comparison with Y-WT mice. However, an opposite effect was found in O-TG mice which exhibited an increase in the number of larger profiles, not only in comparison with O-WT but also when compared with Y-TG mice (Supplemental Fig. 4A).

Regarding the stereological parameters related with mitochondrial abundance, Na was decreased by CYB5R3 overexpression in WF, in both young and old mice (Fig. 5B). Mitochondrial Vv was also decreased by CYB5R3 overexpression in young mice (Fig. 5C), which is in accordance with the decreases of mitochondrial size and Na in Y-TG specimens (Figs. 4B and 5A). While mitochondrial Vv was also decreased with aging in WT mice (Fig. 5C), the strong decrease in mitochondrial size was partially compensated here by an increase in mitochondrial number, which attenuated the diminution of Vv with aging in WT (Fig. 5A–C). Interestingly, despite the decreased number of mitochondrial profiles with aging in TG mice (Fig. 5B), Vv was significantly increased in O-TG (Fig. 5C), not only in comparison with O-WT but also with Y-TG, which is likely due to the significant increase in the size of mitochondrial profiles observed in these animals (Fig. 5A). Representative micrographs of WF cross-sections from all experimental groups are shown in Fig. 5D.

A similar pattern of changes was observed when measurements were carried out in WF longitudinal sections, although changes with age and/or CYB5R3 overexpression were generally found more attenuated in comparison with the changes observed in cross sections (see Supplemental Results and Supplemental Fig. 4C-F).

### CYB5R3 overexpression protect from aging-related alterations of mitochondrial size and abundance in RFs

**Fig. 6:**
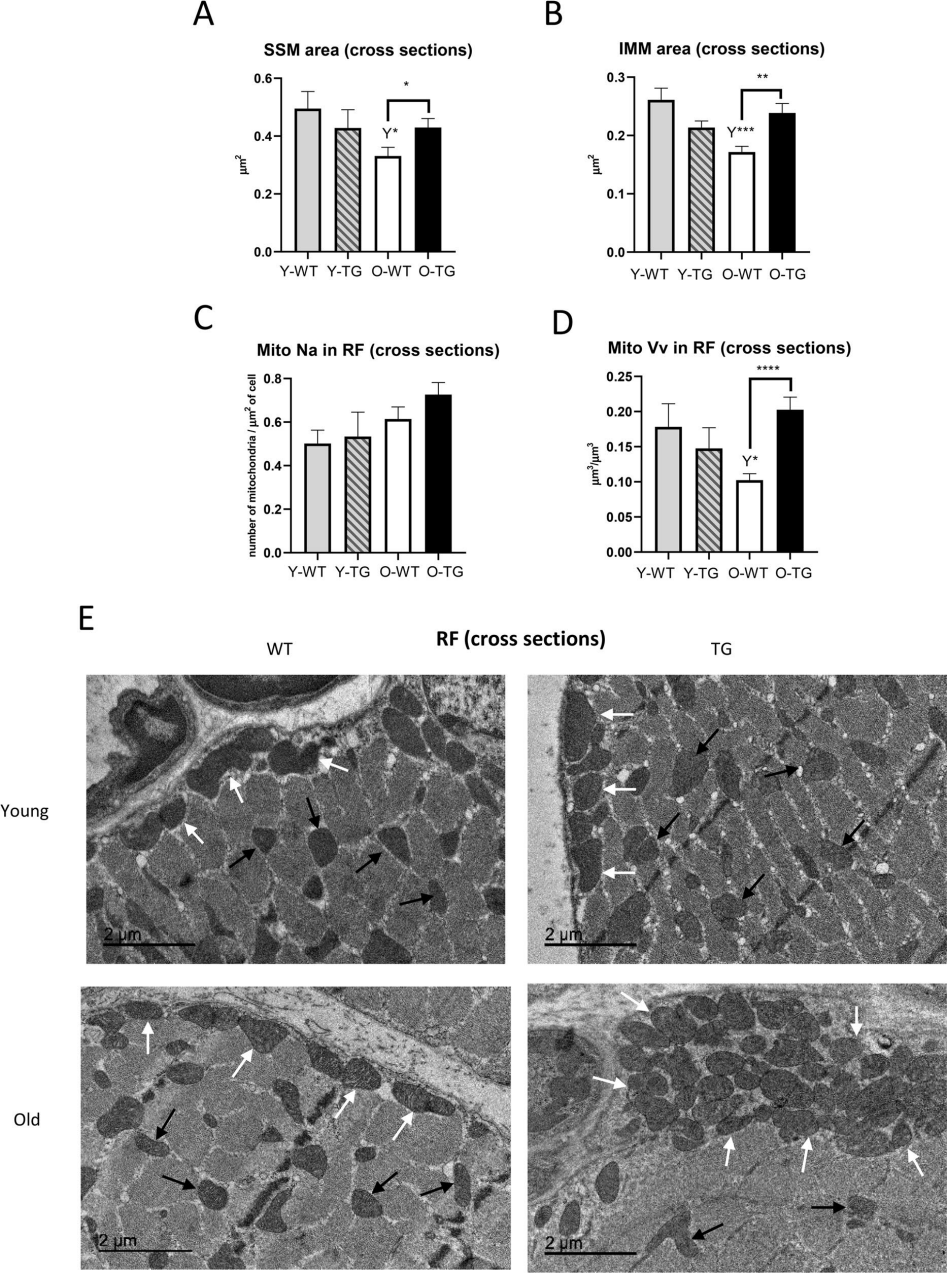
Morphometric characteristics of red fibers (RF) mitochondria in cross sections of gastrocnemius muscle from young-adult and old mice: subsarcolemmal mitochondria (SSM) area (**A**), intermyofibrillar mitochondria (IMM) area (**B**), numerical profile density (Na) (**C**), and volume density (Vv) (**D**). Representative electron microscopy micrographs of each group are shown in **E**. Arrows show subsarcolemmal (white) and intermyofibrillar (black) mitochondria. Asterisks connecting two bars refer to the significance of differences due to CYB5R3 overexpression (WT *vs*. TG) within a given age group. *Y* symbol (accompanied by asterisks) above a bar denotes the statistical significance of differences between age groups (Y *vs*. O) within a given genotype. Data are shown as mean ± SEM of 4 animals

In cross-sections from RF, we found that aging produced a decrease in the mean size of both subsarcolemmal mitochondria (SSM) and intermyofibrillar mitochondria (IMM) in WT mice (O-WT *vs*. Y-WT), but this aging-dependent decrease was blunted by CYB5R3 overexpression in such a way that mitochondrial size was significantly higher in O-TG compared with O-WT mice (Fig. 6A and B). Regarding the mitochondrial stereology in RF, we found that Na remained unchanged among experimental groups (Fig. 6C), but Vv exhibited a trend towards a decrease with aging in WT, most likely due to the decrease in mitochondrial size, but not in TG mice. In consequence, mitochondrial Vv in RF was also significantly higher in O-TG than in O-WT (Fig. 6D). Statistical comparison of size distributions of all measured mitochondrial profiles confirmed the effects of aging, which increased the abundance of smaller SSM and IMM profiles in O-WT compared with Y-WT mice, and of CYB5R3 overexpression which increased the number of larger SSM and IMM profiles in O-TG compared with O-WT mice (Supplemental Figs. 5A and B). In addition, this analysis uncovered a more subtle effect of CYB5R3 overexpression increasing the number of smaller mitochondrial profiles in Y-TG compared with Y-WT, as we had also encountered in WF (see above). Representative micrographs of RF cross-sections from all experimental groups are shown in Fig. 6E.

Most of the alterations highlighted in cross sections were also reproduced in longitudinal sections (see Supplemental Fig. 6A-G). However, profiles of IMM (which were observed smaller with aging in cross-sections) showed no aging-related decrease when measured in longitudinal sections (Supplemental Fig. 6B).

### CYB5R3 overexpression modulates autophagy and mitophagy markers in skeletal muscle

**Fig. 7:**
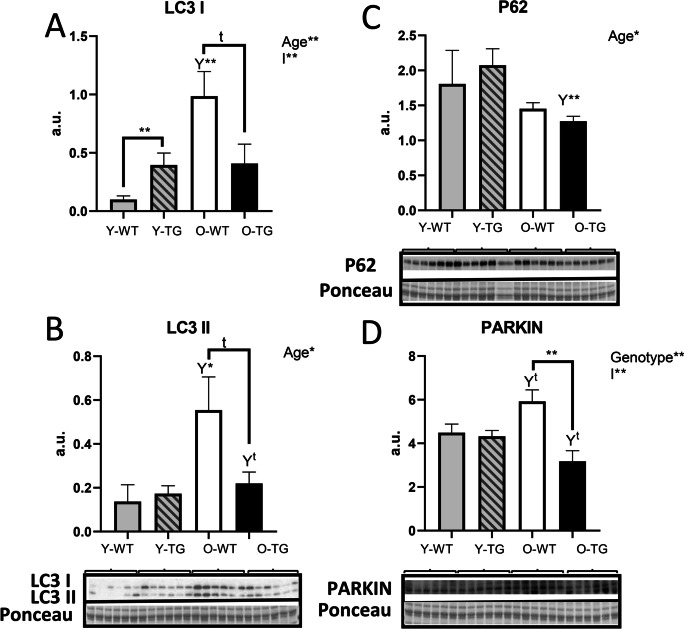
Expression levels of autophagy-related proteins in skeletal muscle of young-adult and old mice of WT and TG genotypes: LC3 A/B I (**A**) and LC3 A/B II (**B**), P62 (**C**), PARKIN (**D**). Asterisks connecting two bars refer to the significance of differences due to CYB5R3 overexpression (WT *vs*. TG) within a given age group. *Y* symbol (accompanied by asterisks or t symbol) above a bar denotes the statistical significance of differences between age groups (Y *vs*. O) within a given genotype. “Genotype” indicates a global effect of CYB5R3 overexpression regardless age, “Age” indicates a global effect of aging independently of genotype, and “I” represents the existence of genotype × age interaction. Antibody- and Ponceau S-stained western blots are included below the corresponding graph (a.u. arbitrary units). Data are shown as mean ± SEM of 6 replicates

Next, we investigated well-established markers of general macroautophagy, as LC3A/B and p62, and of mitophagy, as PINK1 and PARKIN. CYB5R3 overexpression led to a significant increase of LC3A/B I levels in young-adult mice (Fig. 7A), and, although LC3A/B II levels were unaffected (Fig. 7B), these changes were not translated into a significant modification of the LC3A/B II to total LC3A/B ratio (Supplemental Fig. 7A). Of note, aging produced substantial increases of both LC3A/B I and LC3A/B II levels in WT mice, and these increases were blunted by CYB5R3 overexpression (Fig. 7A and B). Again, these changes took place without significant alterations in the LC3A/B II to total LC3A/B ratio (Supplemental Fig. 7A). In the case of p62, our most prominent finding was its substantial decrease in O-TG when compared with Y-TG mice (Fig. 7C). Regarding the two mitophagy markers analyzed, no differences among any of the experimental groups were found for PINK1 (Supplemental Fig. 7B), but a significant decrease in PARKIN was observed in O-TG group compared with O-WT (Fig. 7D).

## Discussion

CYB5R3 plays a key role in the regulation of respiratory metabolism and aging [15]. To gain new insights in this role, we generated TG mice overexpressing CYB5R3, which not only exhibited some of the salutary effects seen with CR, but also lived longer than their wild-type counterparts [16]. A liver transcriptomic analysis of WT and TG mice highlighted significant differences in gene sets related to mitochondrial function. In addition, Ingenuity Pathway Analysis depicted a robust CYB5R3-inducible expression of transcripts associated with aerobic respiratory pathways (coenzyme Q biosynthesis and oxidative phosphorylation) [16]. However, CYB5R3 overexpression hindered some of the metabolic adaptations to CR in hind limb skeletal muscle, particularly the increases of mitochondrial mass and mitochondrial fusion (MFN-1) and biogenesis (NRF-1) markers. Interestingly, TG mice on CR did have a clear induction on TFAM and mitochondrial complex expression [14].

CYB5R3 was highly overexpressed in skeletal muscle homogenates from TG mice, in agreement with our previous studies focused on young-adult animals [14, 16]. Moreover, we show here for the first time that aging does not modify this overexpression pattern, and overexpressed CYB5R3 is efficiently targeted to skeletal muscle mitochondria. This tissue thus emerges as a suitable model to study the direct effects of CYB5R3 overexpression. Endogenous CYB5R3 was significantly upregulated by aging, which agrees with the results reported for human skeletal muscle [24], although levels of the endogenous polypeptide that were reached in old WT mice were still extremely low in comparison with those of TG mice.

Mitochondrial dysfunction is one of the hallmarks of aging [3], while the optimization of mitochondrial function is related with decreased oxidative stress and extended longevity [25]. Representative protein markers of mitochondrial complexes I, III, IV, and V decreased with aging in skeletal muscle from WT mice. In accordance, mitochondrial DNA copy number and the levels of mitochondrially encoded transcripts of cytochrome *c* oxidase I and III were significantly reduced in gastrocnemius of aged male Fischer 344 rats [26], and a decrease of complex IV has been described for aged human skeletal muscle [27]. The age-related decrease of complex I also agrees with previous research from our group [19] and others [6]. However, in our previous studies, we did not observe the aging-related decreases of complexes III, IV, and V we have shown here, which is likely due to differences in study design in terms of age (21 *vs*. 24 months), diet (AIN-93G *vs*. AIN93-M), and feeding pattern (5% of dietary restriction in the control group vs. ad libitum intake in this study).

CYB5R3 overexpression profoundly affected the abundance of ETC complexes, and the increase of complex II in young-adult TG mice also agrees with our previous report [14]. We now show that the most striking effects of CYB5R3 overexpression on ETC complexes are however observed in old TG mice, which exhibited significant increases of complexes I, II, and IV. Furthermore, CYB5R3 overexpression abated the aging-related decreases of complexes I, III, IV, and V, indicating a protective effect against aging-related mitochondrial dysfunction [16]. Of note, overexpression of the skeletal muscle- and heart-enriched lncRNA, LINC00116, which encodes the highly conserved microprotein Mitoregulin (Mtln), promotes a switch from glycolysis to respiration, and enhances respiratory efficiency and optimizes mitochondrial metabolism through a mechanism that relies on its interaction with CYB5R3, which is required to provide a favorable lipidic environment that preserves the interaction of respiratory complex I into supercomplexes [28].

Increased abundance of ETC complexes could be due both to alterations in mitochondrial membrane composition and to changes in mitochondrial abundance. To distinguish between these two possibilities, we measured the levels of VDAC, a well-established biochemical marker of mitochondrial abundance [29]. Mitochondrial mass is known to decrease during aging [30], but we found no changes in the levels of VDAC that could be attributable to either age or to genotype. Nevertheless, maintenance of VDAC with aging has been also documented in skeletal muscle of rhesus monkeys [31]. Therefore, the increases of ETC markers we observe in O-TG mice are apparently not due to changes in mitochondrial abundance, but more likely to intrinsic alterations in the composition of mitochondrial membranes. Likewise, Mtln overexpression also enhances respiratory efficiency by bolstering protein complex assembly and/or stability independent of alterations in mitochondrial mass [32].

Mitochondrial biogenesis declines with aging, and previous studies have demonstrated that TFAM and NRF-1 protein levels decrease in liver from aged rats [33]. Our results have shown that the same is applicable to mouse skeletal muscle although these changes were not prevented by CYB5R3 overexpression. Likewise, the levels of PGC-1α/β, a master regulator of mitochondrial biogenesis, were found unchanged in liver from adult TG mice in comparison with their WT counterparts [16]. On the other hand, CYB5R3 activity sensitizes soluble endothelial guanylate cyclase (sGC) to NO in vascular smooth muscle cells by reducing its heme iron, and CYB5R3 has been recognized as a critical regulator of cGMP production in endothelial cells [34]. cGMP can regulate mitochondrial biogenesis in various cell types and tissues, including mouse gastrocnemius through increased expression of PGC-1α, NRF-1, and TFAM [35, 36]. Since CYB5R3 overexpression does not alter the abundance of PGC-1α polypeptide in liver [16], nor prevent the aging-dependent decline of NRF-1 or TFAM in skeletal muscle (this work), it remains for further investigation to elucidate if the CYB5R3/sGC axis plays a role in regulating the function of skeletal muscle mitochondria along aging through mechanisms independent from mitochondrial biogenesis.

While some studies have indicated that coenzyme Q declines in most of the organs during aging, the changes observed in skeletal muscle coenzyme Q with advancing age have not been uniform. A previous study reported no changes in coenzyme Q levels in whole gastrocnemius homogenates from old in comparison with young mice, although a CR intervention based on every-other-day feeding procedure and physical exercise was able to increase skeletal muscle coenzyme Q levels in old but not in young mice [37]. However, in another work focused on mitochondria isolated from mouse heart, skeletal muscle, kidney, and brain, it was found that coenzyme Q content declined with age specifically in the skeletal muscle, while CR increased coenzyme Q_9_ in skeletal muscle mitochondria [22]. In our model, we found that aging produced a decrease in skeletal muscle coenzyme Q levels (particularly coenzyme Q_9_), which was not affected by CYB5R3 overexpression. Our previous study focused on liver from adult TG mice also reported that hepatic coenzyme Q_9_ levels are not affected by CYB5R3 overexpression [16].

While the aging-related decline of mitochondrial biogenesis markers and coenzyme Q was not prevented in TG mice, CYB5R3 overexpression did avoid the decrease in mitochondrial complexes observed in aged WT mice (see above), which could contribute to maintain the functionality of this organelle with advanced age. ATP content and production have been reported to decline with aging in rat gastrocnemius [38] and in human quadriceps [39]. We thus measured ATP content in skeletal muscle samples from our experimental groups and found a significant reduction with aging in hind limb skeletal muscle from WT mice, which is in accordance with these previous investigations. Remarkably, the decline of ATP content with aging was prevented in TG mice. We reported previously that CYB5R3 overexpression produced a 2.5-fold increase of ATP levels in liver from adult TG mice [16]. Since CR has revealed to be ineffective in protecting against the aging-related decline of ATP in skeletal muscle [38], our findings reinforce the idea that CYB5R3 overexpression exhibits antiaging effects that can proceed through mechanisms that divert, at least partially, from those of CR [14, 16].

Salutary effects of CYB5R3 on metabolism and in the regulation of several pathways that are associated with healthy aging have been explained partially on the increase in NADH oxidation by CYB5R3 activity, which is linked to the activation of the sirtuin family of NAD^+^-dependent histone deacetylases [16]. Among the seven sirtuins that are expressed in mammals, SIRT1 and SIRT3 emerge as key elements regulating mitochondrial function in skeletal muscle [11]. The levels of SIRT-1 tended to increase with aging in WT mice, whereas CYB5R3 overexpression provoked a moderate decrease of SIRT-1 in aged mice. SIRT1 levels have been also reported to increase with aging in rats, while exercise training significantly increased its relative activity [40]. The activity of SIRT-1 decreases in skeletal muscle from aged mice due to a lower content of intracellular NAD^+^, which impairs muscle performance in response to exercise [41]. Interestingly, whereas SIRT-1 promotes proliferation of myoblast precursors, it delays their maturation into myotubes, which suggests an overall negative impact on muscle repair [42]. It remains for further investigation if the changes in SIRT-1 expression levels with aging and/or CYB5R3 overexpression we have reported here might unveil a previously unrecognized role for CYB5R3 in skeletal muscle repair.

SIRT-3 plays a prominent role in the regulation of mitochondrial function by controlling the acetylation state of many mitochondrial proteins, and its expression is highly enriched in metabolic tissues as skeletal muscle, particularly in oxidative type I muscles [43, 44]. In our model, the abundance of the cleaved (mitochondrial) isoform of SIRT-3 was significantly increased in hind-limb skeletal muscle from aged WT mice, and a trend towards an increase due to CYB5R3 overexpression was also observed in young-adult mice, followed by the stabilization of SIRT-3 abundance in old TG mice. Whereas an increase of SIRT-3 in Y-TG mice agrees with the outcome of other antiaging interventions as CR [43, 44], our results with aged mice are apparently in contrast with the reported decrease of SIRT3 protein levels in quadriceps from 26- *vs*. 6-month-old mice fed a standard diet [45], and the decrease also in quadriceps (vastus lateralis) from healthy older (59–76 years old) *vs*. healthy young (18–30 years old) volunteers [46]. It is however important to consider that SIRT-3 levels are strongly dependent on the type of skeletal muscle under study and are also under strong regulation in this tissue by diet and exercise, being dramatically increased after exercise training, fasting, and CR while decreased by high fat feeding [44, 46]. Since O-WT mice were characterized by a generalized decrease of mitochondrial complexes, coenzyme Q, and mitochondrial biogenesis markers, we can argue that increased SIRT-3 levels could represent a compensatory response as an attempt to preserve mitochondrial functionality with aging under our experimental conditions.

Fusion and fission processes are necessary to maintain healthy mitochondria in skeletal muscle with aging [8]. We thus focused our studies towards elucidating the effect of aging and/or CYB5R3 overexpression on mitochondrial dynamics markers, and these analyses were combined with the study of mitochondrial morphology by electron microscopy. In WT mice, aging produced a substantial decrease of MFN-2 that was accompanied by a generalized increase of fission markers. It is striking that CYB5R3 overexpression resulted in similar changes in young/adult TG mice, although no further alterations were produced with aging. Our results fully agree with the previous demonstration that MFN-2 levels decrease with aging in mouse skeletal muscle, leading to mitochondrial dysfunction and the accumulation of damaged mitochondria. Moreover, MFN-2 ablation generates a gene signature linked to aging, characterized by inhibition of mitophagy and impairment of mitochondrial quality, which contributes to exacerbate age-related mitochondrial dysfunction [47]. A lack of MFN-2 decrease with aging in TG mice reinforces the importance of CYB5R3 expression to prevent aging-associated mitochondrial dysfunction in skeletal muscle. We also reported that MFN-2 is increased in hind limb skeletal muscle from CR mice [19]. However, previous research focused on elucidating MFN-2 changes with aging in human skeletal muscle has yielded conflicting results. Whereas some studies have reported a decrease of MFN-2 with aging in human skeletal muscle [27, 48], and its levels were restored by exercise [48], others have reported either similar [49–51] or even higher MFN-2 protein content in aged human skeletal muscles [52], pointing out to the existence of species-specific mechanisms that determine how MFN-2 levels are altered with aging. Mitochondria-bound DRP-1 also increased significantly in TG mice, which agrees with our previous demonstration of higher DRP-1 expression levels in mice fed long-term CR [19].

Aging differentially affects mitochondrial size, depending on the muscle type: whereas soleus (a red, oxidative muscle) displayed fragmented SSM and IMM with aging, and this change was attenuated by CR, white gastrocnemius (a glycolytic muscle) contained enlarged SSM and more complex/branched IMM mitochondria with aging, and CR only had a marginal effect [53].

Both aging and CYB5R3 overexpression led to increases of fission markers in hind limb skeletal muscle following a pattern that, interestingly, resembled closely the changes that had been observed for cleaved SIRT-3. However, aging and CYB5R3 overexpression had different outcomes on mitochondrial morphology and abundance. In cross sections of WF from red gastrocnemius, we found that the increase of fission markers associated with aging was accompanied by decreased mitochondrial size and augmented mitochondrial number in WT mice. In RF, aging also led to decreased size of both SSM and IMM, indicating a prevalence of mitochondrial fragmentation. These findings agree with the changes observed in soleus [53]. In contrast, mitochondrial content in white gastrocnemius was found unchanged by aging [6, 53].

CYB5R3 overexpression also increased the abundance of fission markers and decreased mitochondrial size in WF from young/adult TG mice. However, unlike what it was found with aging, mitochondrial abundance (estimated from both Na and Vv) significantly decreased in skeletal muscle form TG mice, suggesting that fission is accompanied here by enhanced organelle clearance. With advancing age, we found that the number of mitochondria (Na) did not change in TG mice, but their size was increased significantly leading also to higher Vv values. In RF, increased size of SSM and IMM was also observed in O-TG in comparison with O-WT, which also resulted in higher Vv. A lower mitochondrial content accompanied by increased size of the organelle has been also observed in red gastrocnemius from mice fed under CR [19], indicating that these alterations could represent common features of oxidative muscles in mice subjected to these antiaging interventions. On the other hand, it is noteworthy that ultrastructural changes with age and/or CYB5R3 overexpression were generally found more attenuated in longitudinal sections, when compared with the changes observed in cross sections, suggesting that aging and CYB5R3 overexpression might produce subtle alterations in mitochondrial shape.

A higher mitochondrial size in old TG mice cannot be explained by a predominance of fusion *vs*. fission because of the decrease of fusion (MFN-2) and the coordinated increases of all fission markers (DRP-1, FIS1, and MFF). Nevertheless, mitochondrial fission facilitates the degradation of damaged mitochondrial by autophagy [54]. The maintenance of a proper levels of autophagy is crucial to remove damaged organelles, and results showing impaired autophagy during aging in skeletal muscle have been reported [55]. The ratio between LC3 I and II, a marker of autophagic flux, has been reported to not change with aging [19], which agrees with our results. However, we evidenced a dramatic increase of LC3 A/B I and II levels in old WT mice, which could represent their accumulation due to an impairment of autophagy caused by aging [56]. Of note, the levels of LC3 A/B I and II decreased dramatically in old mice overexpressing CYB5R3, which also exhibited decreased levels of p62 and PARKIN. Since increased p62 has been related with reduced autophagic flux during aging in skeletal muscle [19], these changes are compatible with the enhanced consumption of these proteins. We also found decreased p62 in mice fed CR for 18 months, suggesting a possible unblocking of the autophagy flux [19], as we have encountered in old mice overexpressing CYB5R3. Higher autophagic rates in these animals could explain why the activation of mitochondrial fission results in a reduction in the size of the organelle without augmentation of number, and the improvement of autophagic recycling could lead to the preservation of mitochondria with a higher cross-sectional area.

In summary, we show here that CYB5R3 overexpression is not impaired by aging in skeletal muscle from transgenic mice. Whereas its overexpression does not prevent the decrease in mitochondrial biogenesis markers or coenzyme Q with aging, it prevents mitochondrial dysfunction and the decline in autophagy markers. It is important to consider that the studies reported here were conducted on male mice. Given the role of sex in determining the outcome of antiaging interventions, as CR [57], future studies on female mice are warranted to demonstrate that interventions aimed to increase CYB5R3 activity represent a valuable strategy to counteract the deleterious effects of aging on skeletal muscle.

## Supplementary information

Below is the link to the electronic supplementary material.

Supplementary file1 (PDF 94 KB)

Supplementary file2 (PDF 647 KB)

## Data Availability

The data that support the findings of this study are available from the corresponding author upon reasonable request.
