# Supplementary material for: CYB5R3 overexpression preserves skeletal muscle mitochondria and autophagic signaling in aged transgenic mice
Source: GeroScience. 2022 May 9;44(4):2223–41. doi: 10.1007/s11357-022-00574-8 (PMC9616997; doi:10.1007/s11357-022-00574-8)
Supplement: Supplementary file 1 — Supplementary file1 (PDF 94 KB) [file 11357_2022_574_MOESM1_ESM.pdf]

SUPPLEMENTARY INFORMATION

Supplemental Methods

Generation of transgenic mice overexpressing CYB5R3 and crossings

The rat CYB5R3 gene was cloned into pRC/CMV-rDTD plasmid [1] under the control of the human cytomegalovirus immediate-early promoter and the SV40 poly-adenylation sequences. The construct was cleaved from the cloning vector by digestion with SwaI and NruI restriction enzymes and then microinjected into fertilized C57BL/6J eggs at the University of Michigan Transgenic Animal Model Core Facility (http://www.med.umich.edu/tamc/). Surviving eggs were transferred to pseudopregnant B6D2F1 female mice. Stable incorporation of the construct into the genome was validated as reported previously [2]. Wild-type (WT) and CYB5R3-transgenic (TG) mice were distinguished by PCR genotyping with DNA obtained from tail tissue using the primers CACCAAAATCAACGGGACTT (forward) and AGACCGGGGAGAGTACCACT (reverse) to reveal the presence of the transgene. Amplification of the IL2 gene was carried out as internal control using the primers CTAGGCCACAGAATTGAAAGATCT (forward) and GTAGGTGGAAATTCTAGCATCATCC (reverse).

TG males were crossed with WT females obtained from Charles River (Barcelona, Spain) to establish a colony that was maintained under barrier conditions at 22ºC on a 12-hour light/dark cycle, and with *ad libitum* access to water and food at the Service of Experimentation Animals (SAEX) of the University of Córdoba.

Coenzyme Q extractions and measurements

About 20 mg of skeletal muscle tissue homogenate were resuspended in 90 µl of Hanks' balanced salt solution and then solubilized by adding 10 µl of 10% SDS. Next 200 µl of 95:5 ethanol-isopropanol were added, and the samples were vigorously vortexed. Lipids were recovered by extraction with 500 µl of hexane. The samples were centrifuged for 5 min at 12,500 rpm in an Eppendorf Minispin, and coenzyme Q was recovered within the upper hexane phase. This extraction step was repeated twice and both hexane phases were combined. After evaporation of the solvent under vacuum, the lipid extracts were stored frozen at −80 ºC until use.

HPLC separation was carried out with a Beckman Gold System (Beckman Coulter, USA) using a C18 reverse phase analytical column (4.6 mm × 25 cm, Ultrasphere ODS, 5 µm particle) coupled to a Coulochem

II electrochemical detector (ESA, Chemsfold, MA, USA). Separation was carried out at 1 ml/min with a mobile phase composed of mixture of methanol/isopropanol/1M pH 4.4 ammonium acetate (53:45:2). The analytical cell (ESA, Model 5010) was set at potentials of −500 mV and +300 mV in electrodes 1 and 2, respectively. Lipid extracts were dissolved in 30 µl of methanol, and the sample was then subjected to chemical reduction with 1 µl of a freshly prepared 50 mM sodium borohydride solution just before injection. This step results in the complete reduction of the quinones to their corresponding hydroquinones, which allows their detection at the second electrode of the analytical cell with maximal sensitivity and shortens the chromatography time. The entire procedure was performed at room temperature and under these conditions, retention times were 10-11 min for reduced coenzyme Q_9 and 14-15 min for reduced coenzyme Q_{10}. The area units of hydroquinone peaks were integrated and referred to a standard solution of reduced Q_{10}, and then normalized to the amount of protein of each sample, calculated previously with the Bradford assay.

Ultrastructural quantitative analysis of mitochondria from skeletal muscle fibers

Samples of red gastrocnemius were fixed in a mixture of 2.5 % glutaraldehyde–2 % paraformaldehyde in 0.1 M sodium cacodylate buffer pH 7.2 for 12-24 h and post fixed in 1 % osmium tetroxide for 1 h at 4 °C in the same buffer. The pieces were dehydrated in a series of ethanol's, transferred to propylene oxide and sequentially infiltrated in EMbed 812 resin (Electron Microscopy Sciences, PA, USA). We used the sequence propylene oxide–resin 2:1, 1:1, and 1:2 throughout 24 h (8 h each). Afterwards, samples were transferred to pure resin for 24 h. Then, blocks were formed in silicon molds with fresh resin for 48 h at 65 °C. We placed the samples into the molds to obtain both cross and longitudinal sections of the tissues.

After trimming, blocks were sectioned in an Ultracut Reicher ultramicrotome. We obtained thin sections (40-70 nm thick) that were mounted on nickel grids. After staining in aqueous 4% uranyl acetate and lead citrate (following the instruction of the dealers), the sections were examined and photographed in a Jeol JEM 1400 electron microscope at the Servicio Centralizado de Apoyo a la Investigación (SCAI; University of Córdoba; Spain). Pictures of cross- and longitudinal sectioned samples were taken from both peripheral and internal zones of the cells for different purposes.

Supplemental Results

Ultrastructure, planimetry and stereology of skeletal muscle mitochondria in longitudinal sections from WF

Changes due to aging and/or CYB5R3 overexpression on ultrastructural features of gastrocnemius WF mitochondria in longitudinal sections were similar to those observed in cross sections, although the extent of these changes was found attenuated in comparison with the differences observed in cross sections, particularly in the case of mitochondrial size, which did not differ significantly among experimental groups (Supplemental Figure 4C). Here, the most prominent change was the decrease of Vv (Supplemental Figure 4E), most likely due to a similar decrease in mitochondrial number in Y-TG compared with Y-WT mice (Supplemental Figure 4D), although CYB5R3 overexpression did not changed these parameters in old mice. Distribution analysis of the size of all mitochondrial profiles measured in longitudinal sections (Supplemental Figure 4B) gave similar results to that of transversal sections (Supplemental Figure 4A) but differences, although indeed statistically significant, were not so conspicuous, suggesting that aging and CYB5R3 overexpression affect the shape of the organelle in WF. Representative micrographs of WF longitudinal sections from all experimental groups are shown in Supplemental Figure 4F.

Ultrastructure, planimetry and stereology of skeletal muscle mitochondria in longitudinal sections from RF

Analysis of longitudinal sections confirmed the decrease of SSM size with aging in WT mice (Supplemental Figure 6A). However, IMM which were found smaller in cross-sections showed no decrease in longitudinal sections (Supplemental Figure 6B), indicating again a modification in the shape of these organelles with aging. Increase of mitochondrial size (both SSM and IMM) by CYB5R3 over-expression in old mice was also confirmed (Supplemental Figures 6A and B). Regarding the stereological parameters we also confirmed the increase in mitochondrial volume density (Vv) in old mice overexpressing CYB5R3 (Supplemental Figure 6D), but without statistically significant changes in mitochondrial number (Na, see Supplemental Figure 6C). Distribution analysis of the size of all mitochondrial profiles measured in longitudinal sections showed again that the effect of CYB5R3 overexpression on mitochondrial size is maximized in old animals (Supplemental Figure 6E and F). Representative micrographs of RF longitudinal sections from all experimental groups are shown in Supplemental Figure 6G).

Supplemental Figure legends

Supplemental figure 1. Expression levels of CYB5R3 polypeptide in whole extracts (A) and in enriched mitochondrial fractions (B) isolated from hind limb skeletal muscle from young and old mice of WT and TG genotypes. The figure depicts the same Western blots as those shown in Figures 1A and 1B, but images were obtained after over exposition in the quimioluminiscence reaction to reveal quantitatively the signal from WT mice while saturating that from TG mice (seen as red bands in the western blots). In all graphs, asterisks connecting two bars refer to statistically significant differences as a function of age in mice of the WT genotype. Since the signal from TG was saturated, no quantification was performed here. Depicted data are mean ± SEM of 6 replicates. Western blots and Ponceau S staining lanes are included below the graph (a.u. = arbitrary units).

Supplemental figure 2. Total coenzyme Q (coenzyme Q_9 plus coenzyme Q_{10}) as measured by HPLC (A), and abundance of full-length SIRT3 polypeptide as measured by western blot (B) in skeletal muscle homogenates (antibody- and Ponceau S-stained western blots are included below the graph). A "t" symbol connecting two bars refer to the significance of differences due to CYB5R3 overexpression (WT *vs*. TG) within a given age group. *Y* symbol (accompanied by asterisks) above a bar denotes the statistical significance of differences between age groups (Y *vs*. O) within a given genotype. "*Age*" indicates a global effect of aging independently of genotype, and "*I*" represents the existence of *Genotype* x *Age* interaction. Data are shown as mean ± SEM of 6 replicates (a.u. = arbitrary units).

Supplemental figure 3. Expression levels of MFN-1 (A), DRP-1 measured in enriched mitochondrial fractions (B) and VDAC in enriched mitochondrial fractions too (C) in skeletal muscle of young and old WT and TG animals. Asterisks connecting two bars refer to the significance of differences due to CYB5R3 overexpression (WT *vs*. TG) within a given age group. "*Age*" indicates a global effect of aging regardless genotype. "*I*" represents the existence of *Genotype* x *Age* interaction. Data are shown as mean ± SEM of 6 replicates. Western blots are included below the graph (a.u. = arbitrary units).

Supplemental figure 4. Planimetric (area) and stereological features of WF mitochondria. Panels A and B show the size distribution of mitochondria in cross and longitudinal sections respectively. Mean area (C), numerical profile density (Na) (D), and volume density (Vv) (E) of mitochondria in WF longitudinal sections are also shown. Representative electron microscopy micrographs of each group are shown in panel F. Some examples of mitochondria are shown with arrows. Asterisks or "t" symbol connecting two groups

refer to statistically significant differences between these groups. *Y* symbol (accompanied by "t" symbol or by asterisks) above a bar denotes the statistical significance of differences between age groups (Y *vs*. O) within a given genotype.

Supplemental figure 5. Size distribution of RF mitochondria in cross sections: SSM (A) and IMM (B). Asterisks connecting two groups refer to statistically significant differences between these groups.

Supplemental figure 6. Planimetric (area) and stereological analyses of mitochondria in longitudinal sections of RF: subsarcolemmal mitochondria (SSM) area (A), intermyofibrillar mitochondria (IMM) area (B), numerical profile density (Na) (C), and volume density (Vv) (D). Panels E and F show the size distributions of SSM and IMM in longitudinal sections of RF. Panel G shows representative electron microscopy micrographs of each group. Some examples of subsarcolemmal and intermyofibrillar mitochondria are shown with white and black arrows, respectively. Asterisks or "t" symbol connecting two groups refer to statistically significant differences between these groups. *Y* symbol (accompanied by "t" symbol or by asterisks) above a bar denotes the statistical significance of differences between age groups (Y *vs*. O) within a given genotype. Data are shown as mean ± SEM of 4 animals.

Supplemental figure 7. Expression levels protein related to autophagy in skeletal muscle from young-adult and old mice of WT and TG genotypes: LC3A/B II to total LC3A/B ratio (A), and PINK1 (B). Antibody- and Ponceau S-stained western blots are included below B.. Data are shown as mean ± SEM of 6 animals.

Supplemental references

[1] Belcourt MF, Hodnick WF, Rockwell S, Sartorelli AC. The intracellular location of NADH:cytochrome b_5 reductase modulates the cytotoxicity of the mitomycins to Chinese hamster ovary cells. J Biol Chem. 1998;273(15),8875-8881. doi: 10.1074/jbc.273.15.8875

[2] Martín-Montalvo A, Sun Y, Díaz-Ruíz A, Ali A, Gutiérrez V, Palacios HH, *et al*. Cytochrome b_5 reductase and the control of lipid metabolism and healthspan. NPJ Aging Mech Dis. 2016;2,16006. doi:10.1038/npjamd.2016.6
