# Supplementary material for: CYB5R3 overexpression preserves skeletal muscle mitochondria and autophagic signaling in aged transgenic mice
Source: GeroScience. 2022 May 9;44(4):2223–41. doi: 10.1007/s11357-022-00574-8 (PMC9616997; doi:10.1007/s11357-022-00574-8)

Suppl. Figure 1

A

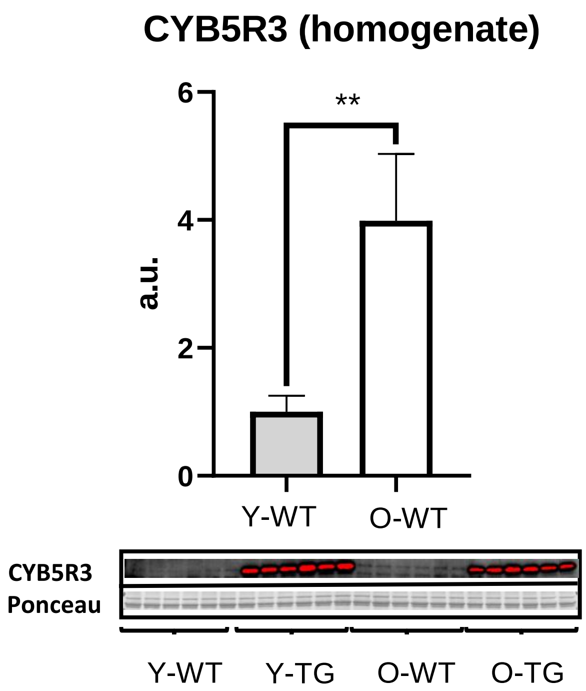


B

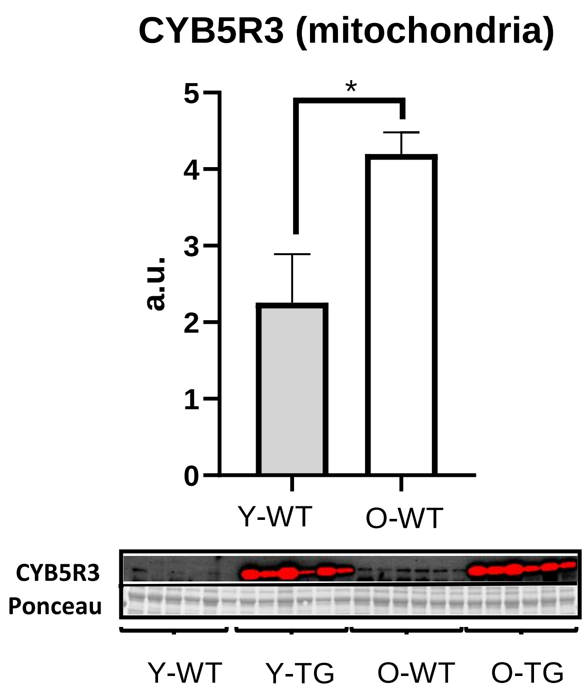

Suppl. Figure 2

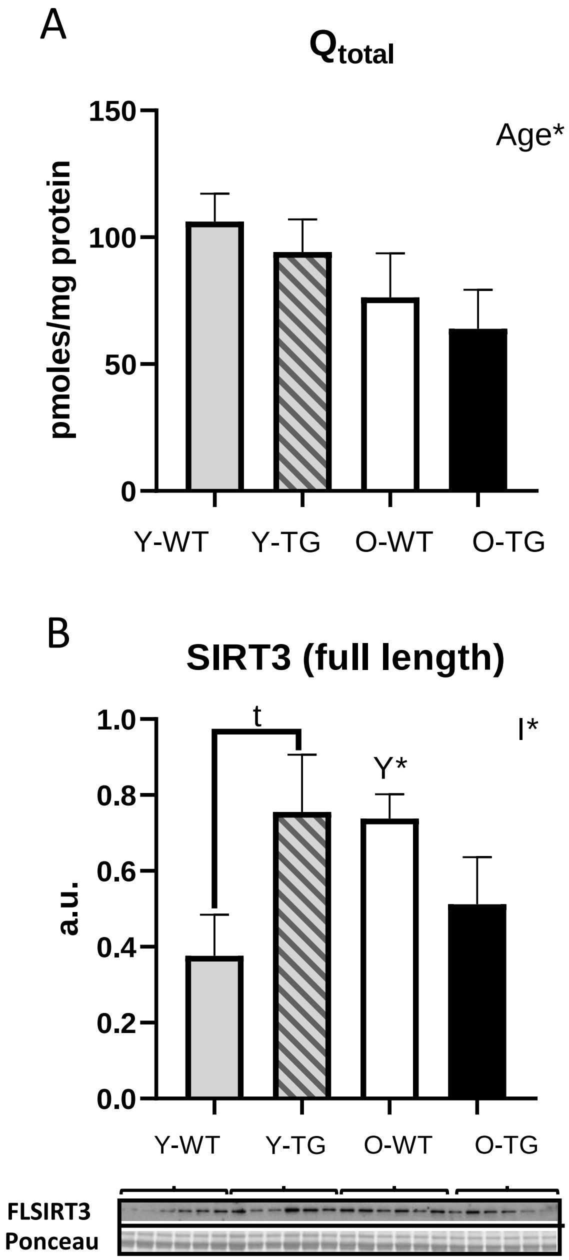

Suppl. Figure 3

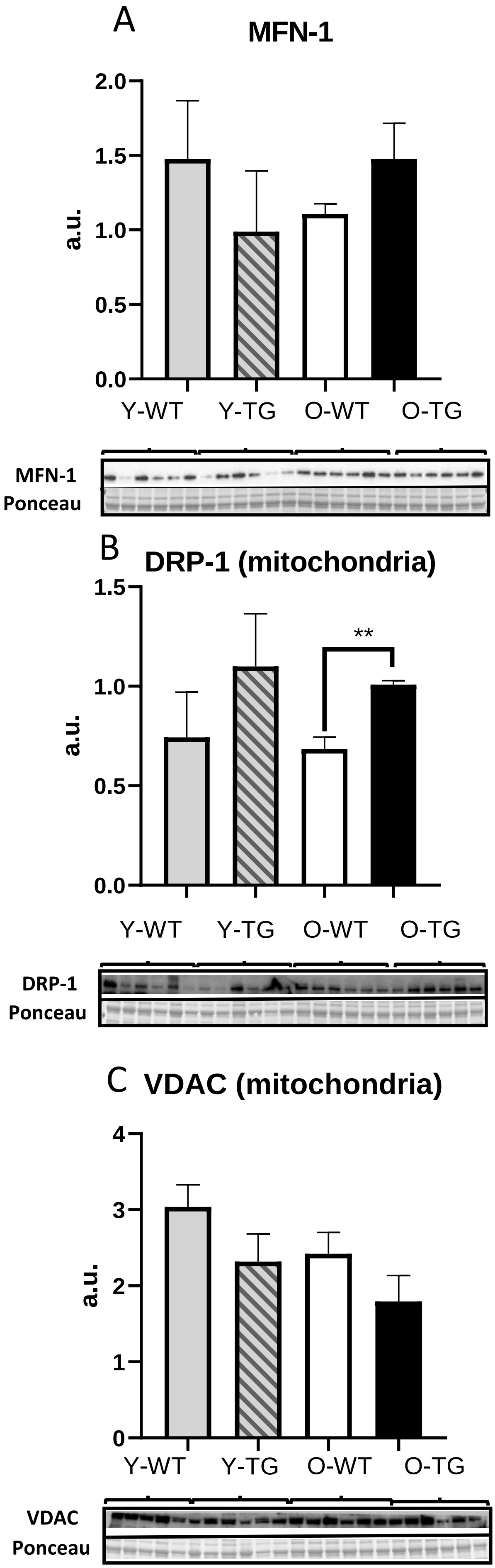

# Suppl. Figure 4

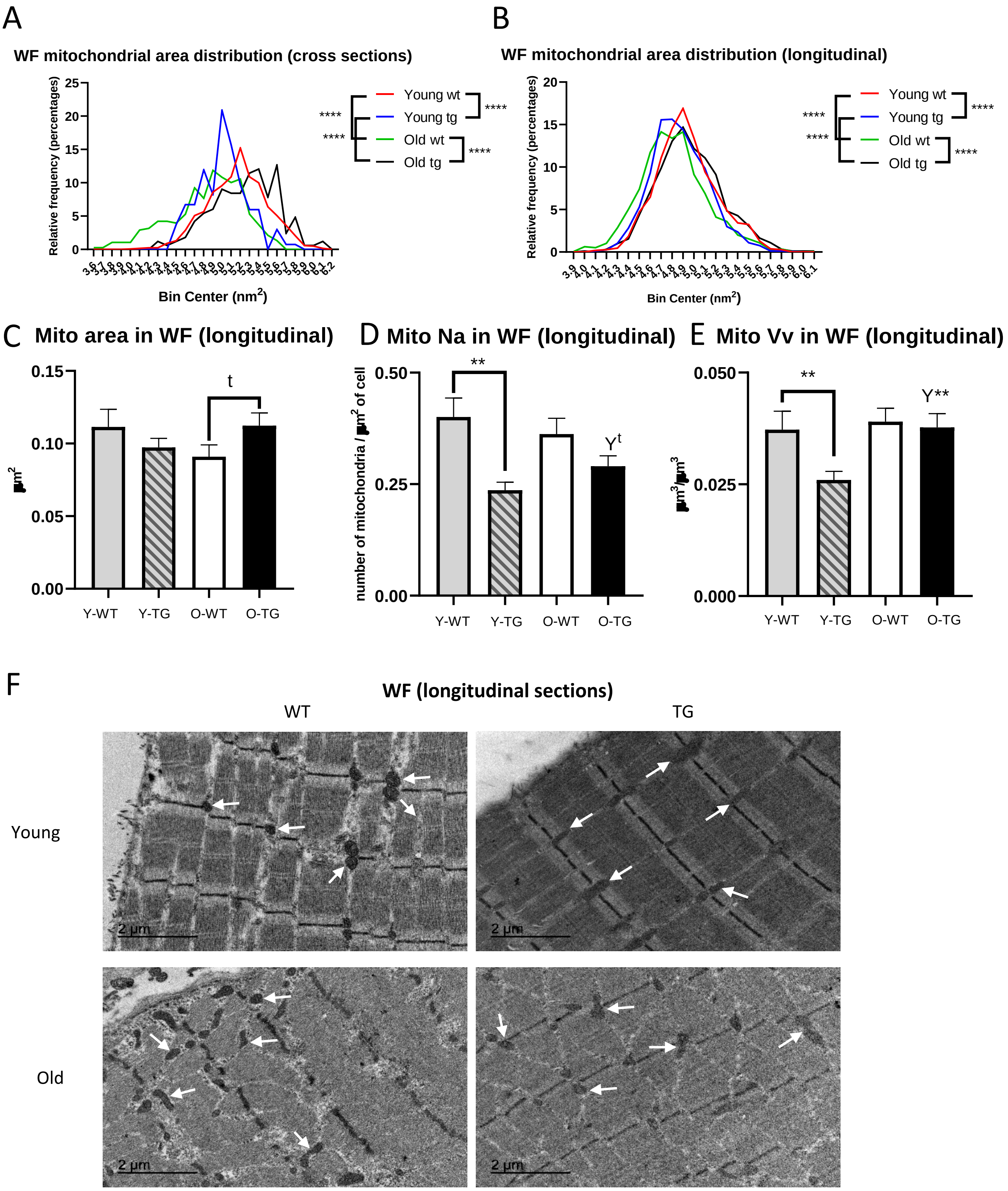

Suppl. Figure 5

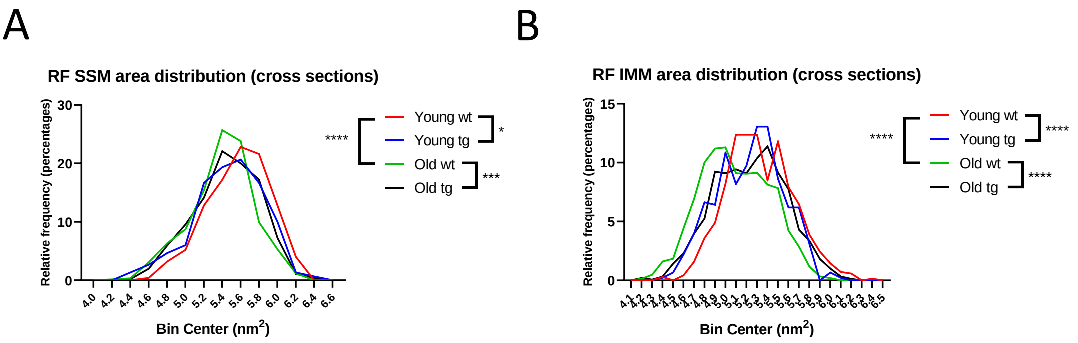

# Suppl. Figure 6

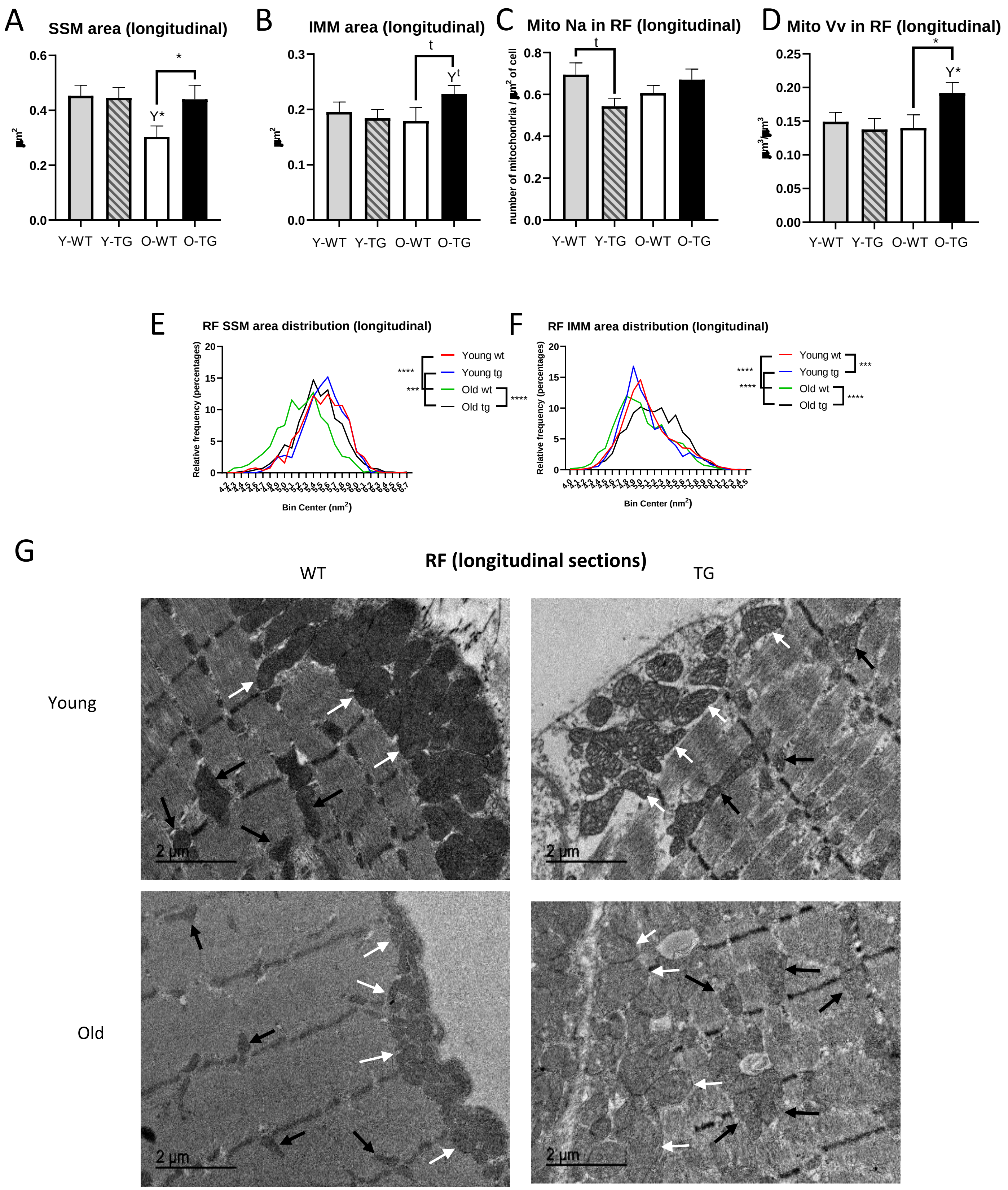

Suppl. Figure 7

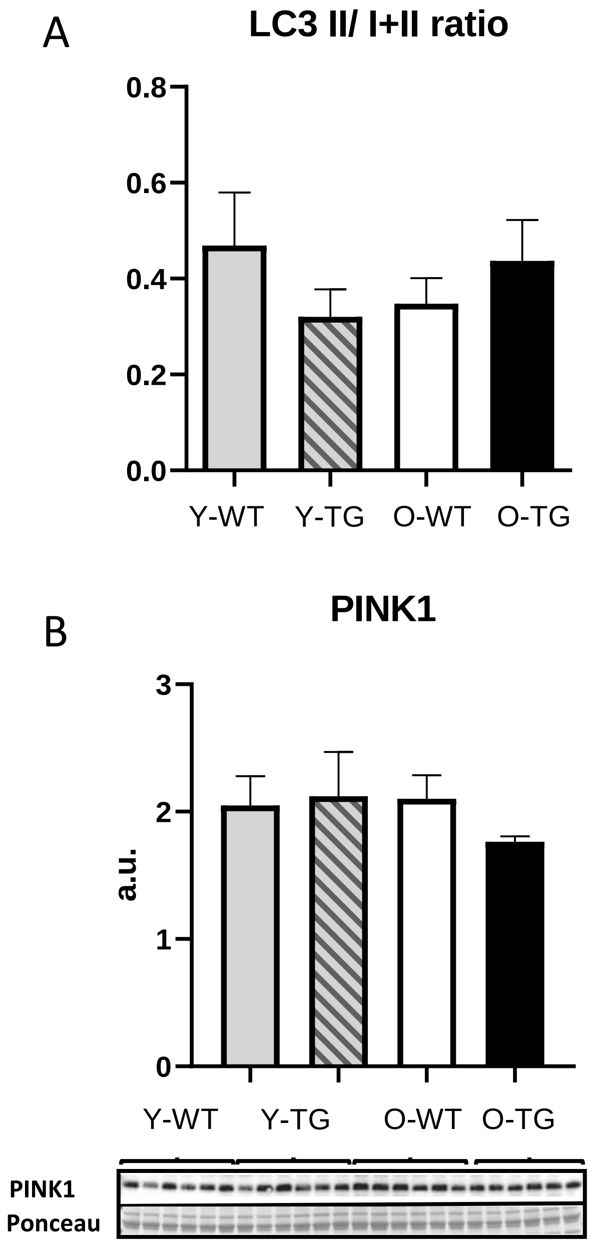

Supplement: Supplementary file 2 — Supplementary file2 (PDF 647 KB) [file 11357_2022_574_MOESM2_ESM.pdf]
